# Enteric methane emission factors of smallholder dairy farming systems across intensification gradients in the central highlands of Ethiopia

**DOI:** 10.1186/s13021-023-00242-0

**Published:** 2023-11-29

**Authors:** Abraham Abera Feyissa, Feyera Senbeta, Adugna Tolera, Dawit Diriba, Kalaya Boonyanuwat

**Affiliations:** 1 College of Agriculture and Natural Resource, Selale University, Fitche, Ethiopia; 2 Department of Biological Sciences Faculty of Sciences, University of Agriculture and Natural Resources, Gaborone, Botswana; 3 School of Animal and Range Sciences, Hawassa University, Hawassa, Ethiopia; 4 Department of Forest Management, University of British Colombia, Vancouver, Canada; 5 Department of Livestock Development, Bureau of Animal Husbandry and Genetics Improvement, Pathum Thani, Thailand; 6 College of Development Studies, Center for Environment and Development, Addis Ababa University, Addis Ababa, Ethiopia

**Keywords:** Enteric methane emission, Emission factor, Emission intensity, Ethiopia, Smallholder dairy farming systems

## Abstract

**Background:**

Following global pledges to reduce greenhouse gas (GHG) emissions by 30% by 2030 compared to the baseline level of 2020, improved quantification of GHG emissions from developing countries has become crucial. However, national GHG inventories in most Sub-Saharan African countries use default (Tier I) emission factors (EF_S_) generated by the Intergovernmental Panel on Climate Change (IPCC) to estimate enteric CH_4_ emissions from animal agriculture. The present study provides an improved enteric CH_4_ emission estimate (Tier II) based on animal energy requirements derived from animal characteristics and performance data collected from about 2500 cattle in 480 households from three smallholder farming systems to represent the common dairy farming in the central highlands of Ethiopia. Using average seasonal feed digestibility data, we estimated daily methane production by class of animal and farming system and subsequently generated improved EF.

**Results:**

Our findings revealed that the estimated average EF and emission intensities (EI) vary significantly across farming systems. The estimated value of EF for adult dairy cows was 73, 69, and 34 kg CH_4_/cow/year for urban, peri-urban, and rural farming systems, respectively. Rural dairy farming had significantly higher emission intensity (EI) estimated at 1.78 CO_2_-eq per kg of fat protein-corrected milk (FPCM) than peri-urban and urban 0.71 and 0.64 CO_2_-eq kg^−1^ FPCM dairy farming systems, respectively. The EF estimates in this study are lower than the IPCC's (2019) default value for both stall-fed high-productive and dual-purpose low-productive cows.

**Conclusions:**

The current findings can be used as a baseline for the national emission inventory, which can be used to quantify the effects of future interventions, potentially improving the country's commitment to reducing GHG emissions. Similarly, this study suggests that increased animal productivity through improved feed has a considerable mitigation potential for reducing enteric methane emissions in smallholder dairy farming systems in the study area.

**Supplementary Information:**

The online version contains supplementary material available at 10.1186/s13021-023-00242-0.

## Background

Enteric methane (CH_4_) is the second largest source of regional greenhouse gas (GHG) emissions from agriculture and represents 14% of all agricultural, forestry, and other land-use emissions [1]. In Africa, agricultural emissions account for the majority of GHG emissions, with livestock production contributing 70% of all emissions, the majority of which are CH_4_ emissions from enteric fermentation [2]. Some estimates show that GHG emissions from enteric fermentation, manure management, and managed soil account for the largest share (60%) of Ethiopia's total agricultural GHG emissions [3]. In addition, methane emissions from enteric fermentation in Ethiopia have shown an increasing trend [4, 5].

Because of the rising demand for animal products, the cattle population is expected to grow, hastening the rise in enteric methane emissions caused by population growth, urbanization, and dietary changes. Given the current demand for livestock products, for instance, the cattle population is likely to increase from today's around 65 million [6] to more than 90 million in 2030 CRGE [7], thereby doubling emissions from the livestock sector. In a business-as-usual scenario, emissions from cattle are projected to increase as a function of cattle population growth, low animal productivity, poor animal health, and low-quality feed, mainly driven by an increase in methane emissions from enteric fermentation.

On the other hand, the dairy value chain provides opportunities for low-cost mitigation and widespread social and economic benefits [8], as there is a strong correlation between increased animal productivity and reductions in enteric methane emissions [9]. There is considerable agreement that increasing efficiency in resource use is a crucial component of improving the sector's environmental sustainability. Improving animal and herd productivity is one of the key pathways to reducing enteric CH_4_ emissions per unit of product [10, 11]. Improved practices and technologies such as strategic supplementary feeding, diet quality, adequate animal health control, and genetic improvement of animals can improve dairy productivity and reduce emission intensity [11].

To contribute to global efforts to reduce methane emissions across all sectors of the economy, the livestock sector must be integrated into national climate action. East African countries, including Ethiopia, have been signatories to the global methane pledge since 2021 and have committed to reducing global methane emissions by 30% by 2030 compared to baseline levels in 2020 [12]. Furthermore, countries have submitted updated national determined contributions (NDCs) covering methane to the United Nations (UN) Framework Convention on Climate Change (UNFCCC) in 2020, with mitigation actions for the livestock sector included in these submissions [13]. The effectiveness of any national or regional mitigation measure depends on the accuracy, transparency, and comparability of country-specific national GHG inventories [14]. In light of this, countries should adopt a high-tier method to generate baseline information that could be an input to the national emission inventory to quantify the effects of future interventions [15]. Three methods for calculating methane emissions have been developed by the Intergovernmental Panel on Climate Change (IPCC), ranging from the simple Tier I to more complex Tier II and Tier III approaches. Tier I approach uses default emission factors and livestock population to estimate enteric methane emissions, which do not account for possible differences in CH_4_ emissions between cattle of different breeds, ages, and physiological states or differences in intake levels and diet compositions. Using a Tier II method for enteric methane emission estimates would, therefore, improve accuracy and reduce uncertainties caused by the IPCC Tier I method [16].

So far, only a few attempts have been made to estimate the enteric methane emissions of smallholder dairy cattle in Ethiopia in general and the central highlands in particular. Selale milkshed is one of the region in the central highlands of Ethiopia where crop-livestock farming is the predominant practice [17]. Some previous studies have either estimated enteric CH_4_ emissions at the regional or national level [6, 11] or used assumed characteristics of typical farming systems and actual data from small samples of farms [18, 19]. As a result, the findings of those studies are highly aggregated and provide little importance in addressing local differences in production characteristics and intervention measures.

Moreover, ruminant livestock in various agro-ecology and production systems have access to different types and quantities of feed, resulting in varying levels of production and GHG emissions [20, 21]. Herrero et al. [20] noted that the spatial distribution of GHG produced by ruminant animals varies significantly depending on their location due to agroecology and the type of production systems. Provided that livestock husbandry practices and feed resources are dynamic and location-specific, animal and feed characteristics data is pertinent to evaluating enteric CH_4_ emissions for further mitigation actions.

Therefore, the objective of the present study was to generate improved enteric methane emission factors (EF) using the Tier II method based on animal energy requirements derived from animal characteristics and performance data and to evaluate the variations in enteric CH_4_ emissions among farming systems in the central highlands of Ethiopia.

## Literature review

### Smallholder farming systems

Like most dairy systems found in the tropics, the smallholder dairy farming systems in Ethiopia can be classified into three categories: urban, peri-urban, and rural. These systems are classified depending on the scale of production, production resources, breeding and marketing objectives, management practices, location, and the contribution of dairying to livelihoods [22, 23]. According to Gizaw et al. [23], variables that significantly contributed to classification included the breeds and genotypes kept, daily milk production, income from livestock, and cow feeding practice. Urban and peri-urban dairy production systems constitute most of the country’s improved dairy stock and might share some similar characteristics. Whereas, the rural dairy production system mainly uses indigenous breeds [23].

#### Urban farming systems

These farming systems are concentrated in major cities and towns. The system is market-oriented, based on improved breeds (crossbreds or high-grade), and operated under stall feeding conditions with little or no land resources. As compared to other systems, they have relatively better access to inputs (e.g., feeds) and services (e.g., artificial insemination) provided by the public and private sectors than others and use intensive management. The primary farm output is fluid milk, which is sold to the most affluent urban markets. Farmers dominantly raise exotic or cross-breed exotic blood with local breeds, and agro-industrial byproducts and purchased roughage are the important sources of feed [23–25].

#### Peri-urban dairy systems

The systems are located mainly near towns and cities. Production is market-oriented and specifically targets consumers in urban areas, and some local butter products are the main production objectives in this system [11, 26]. Farmers have access to land and usually practice mixed crop-livestock farming, which produces part of the feed in the form of crop residues and grazing. Similar to urban dairy, in this system too, milk production, in general, is mainly based on cattle (both cross and highbred) and uses semi-grazing and stall feeding using roughage and agro-industrial by-products. The major sources of feed include hay from private grazing land, concentrates, improved forage, and communal grazing land [23].

#### Rural farming systems

These systems are part of the subsistence farming systems that are primarily concentrated in the highlands and are dominated by cereals and cash crops [22]. The system is traditional and based on low-productive multipurpose indigenous cattle breeds for milk, manure (to fertilize the soil and fuel production), and castrated male animals for draught power [22, 23]. Milk is mostly consumed at home or sold to neighbors because rural farmers have limited access to inputs and services, as well as in the urban market, where fluid milk is demanded [11, 24, 26]. The animals mainly graze natural pastures of nonarable or fallow land between crop fields and are additionally fed crop residues.

A variety of factors impede smallholder dairy production, the nature and magnitude of which vary depending on production systems and agro-ecologies. Some are system-specific, affecting specific dairy production systems regardless of agroecology, whereas others are cross-cutting [22]. Among the major constraints are feed shortages in terms of quality and quantity, the low genetic potential of indigenous breeds, poor access to inputs and services, undeveloped market linkage, land shortages, and policy support for dairy development [22, 23]. In all farming systems, farmers' ability to realize the genetic potential of improved breeds and increase output is limited by non-genetic constraints. Natural pastures, crop residues, and other available forages, for instance, have lower digestible energy and protein content, limiting milk yield and increasing enteric methane emissions [26]. Variations in the livestock management systems (feed quantity and quality, feeding level, and livestock activity and health) also have a wide range of effects on enteric CH_4_ emissions [27, 28]. In light of this, examining variations in enteric methane emissions across smallholder farming systems will be essential to evaluating baseline information used for the national CH_4_ emissions inventory and future climate actions that integrate livestock into global efforts to reduce CH_4_ emissions.

## Methods

### The study area

**Fig. 1:**
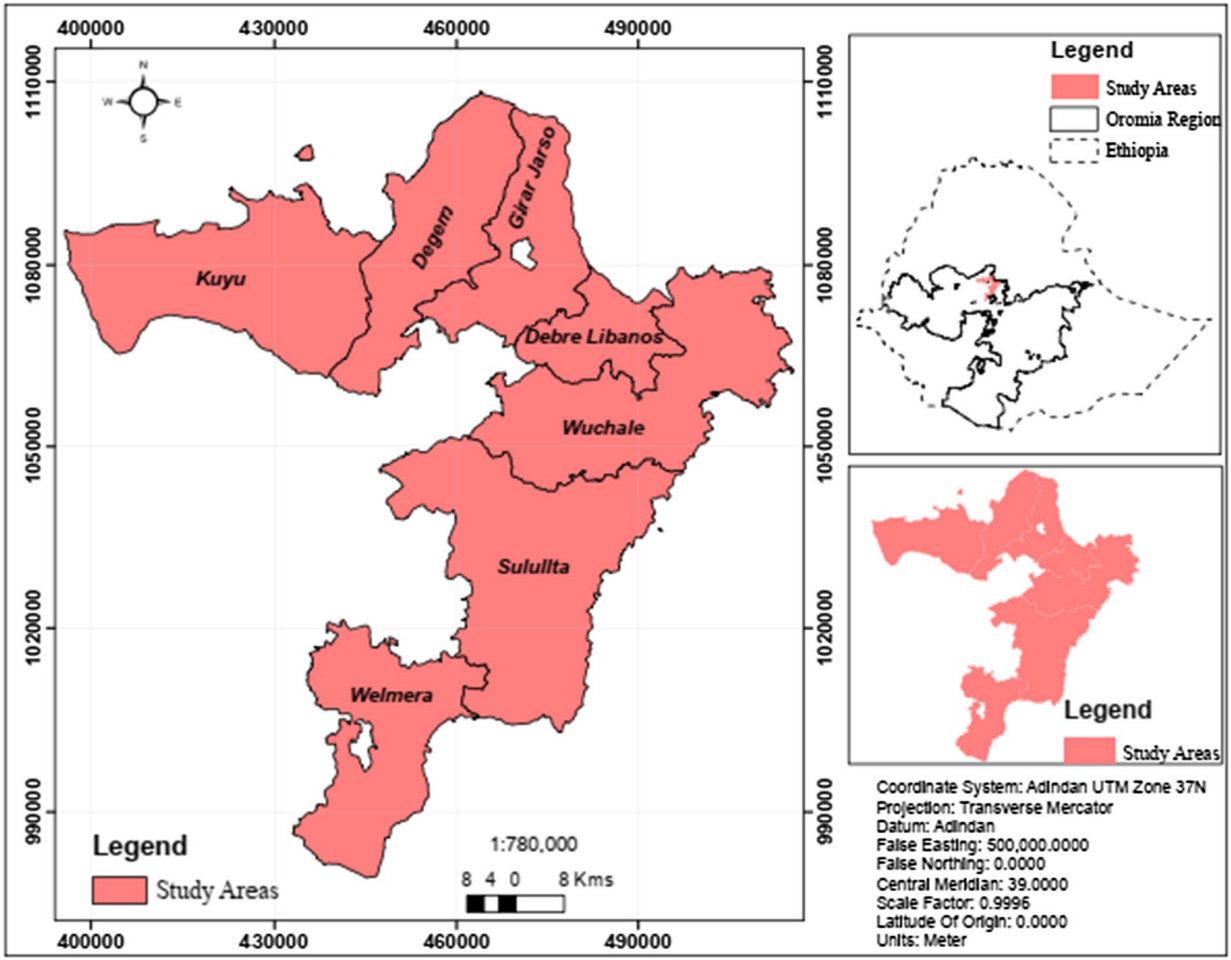
Location map of the study area

This study was conducted in the Selaleshed in the central highlands of Ethiopia (Fig. 1)*.* The study area is located at 38°07′60" E longitude and 9°40′60''N latitude [17]. The area includes diverse topographical features ranging in altitude from 3500 m above sea level (m.a.s.l.) at the tip of the mountainous to 1200 masl across most plains. Average annual rainfall ranges from less than 1400 to 1600 mm, while the mean annual temperature varies between 7.90 and 19 °C [17, 29]. The area receives bimodal rainfall during the summer (June–September) and spring (February–April). The most important agricultural enterprise is livestock, particularly dairy farming, and agricultural production is primarily subsistence mixed crop-livestock farming, with smallholder dairy farms dominating the dairy industry [17].

The study area was chosen because of its high milk production potential and the importance of smallholder dairy in the local subsistence agriculture economy. The Selale milkshed is known to have a high percentage of improved dairy breeds, better access to artificial insemination, and suitable conditions for fodder production and use of agricultural by-products [4, 22, 30]. According to the CSA (2020) report, the area has the largest population of crossbred dairy cows in Ethiopia.

### Sampling techniques

We collected first-hand information about dairy production, feed resources, and related matters through a quick survey and discussions with agricultural extension offices. The information was used to identify focal Kebeles (the smallest administrative unit in Ethiopia) and individual farmers registered under the national dairy cattle database, using a multi-stage purposive sampling technique. Four potential mixed farming districts (Suluta, Wuchale, Girar Jarso, and Degem) were purposively selected to represent the diverse agroecology and smallholder dairy farming systems of the study area. Girar Jarso district was selected to represent urban smallholder farming systems, where dairy farming is practiced to support family income in addition to off-farm activities. Sululta and Wuchale districts represent mid-land agroecology and peri-urban dairy farming systems where crop and livestock production are closely integrated. Degem district is a typical highland agroecology where crop and livestock (dairy) production are closely integrated.

Four kebeles were purposively selected from each district by considering smallholder farmers (SHF) registered under the national dairy cattle database and proximity to the road. Smallholder farmers in each kebele were stratified into urban, peri-urban, or rural dairy farmers based on location, production scale, production resources, feeding system, breeds and genotypes kept, and the contribution of dairy to livelihoods [22, 23]. In the third stage, 30 households (480 in total) were randomly selected from each of the selected kebeles. The study adopted the sample size rule of Arsham [31] for sample size determination and Wilkes et al. [32] for smallholder dairy methodology.

### Data collection

This study relied on farm household surveys, activity data from national dairy cattle databases, and secondary sources.

#### Household survey

A household survey was carried out between July 2020 and February 2021. The SHFs were visited three times to observe seasonal variations in feed resources and cross-check the data. Under the close supervision of the first author, a team of enumerators with expertise in livestock production administered the questionnaire to randomly selected household heads. The questionnaire was designed to obtain information on household characteristics, livestock holding, dairy cattle herd management, reproductive and production performance, major feed available in the area and feeding system, disease prevalence, marketing, and major production constraints. Farmers and village leaders participated in 6–8 person focus group discussions to validate the information gathered through individual farmer interviews. Besides, secondary data on human and livestock populations, feed chemical composition, and climate were gathered from zonal and district agriculture and rural development offices.

### Animal characteristics and performance data

#### Herd composition

For this study, a total of about 3000 cattle of various ages and sexes were used (Table 3). Then the animals were classified into four age groups: adult animals (3–10 years); growing animals (1–3 years); calves (6 months-1 year); and male and female calves (< 6 months). To refine this information further, we traced back to the owner's profile and individual cattle records for more details, and animals that were culled or missing during the farm visit were excluded. The cattle population comprises pure Holstein Friesian, East African shorthorn zebu, and their crosses. In the urban and peri-urban farming systems, pure Holstein cattle and their crosses are kept, while in the rural farming system, indigenous cattle (East African shorthorn zebu breed) are kept.

### Live weight measurement and average daily weight gain

Live weight (LW), mature weight, and average daily weight gain data were extracted from the national dairy cattle database found in the Livestock Development Institute (LDI). The LW of animals was then estimated from the heart girth measurements using the regression equation by Goopy et al. [33]. Due to the unreliability of our calculated value, we relied on secondary sources [34, 35] for average daily weight gain (ADG). Body condition score (BCS) was subjectively assessed and scored using the 5-point scale 1–5 scale by following Edmonson et al. [35].

#### Milk yield and its chemical composition

Data for milk yield and reproductive performances (age at first calving, calving interval, calving date, milk yield per day, milk test date, and dry date) were extracted from the national dairy cattle database. The standard 305-d milk yield for each animal was estimated from test date (TD) milk yield records by using a test interval method described by Sargent et al. [36] and Migose et al. [37]. Milk samples were collected from a total of 70 SHFs (urban = 27, peri-urban = 21, and rural = 21) in the morning and afternoon and analyzed for chemical composition such as butterfat (% BF) (Table 1) [38], by the Gerber method, and milk density [39]. Richmond's formula [40] was used to calculate milk solid not-fat (SNF) as follows:

**Table 1:** Chemical composition of milk in the study area

Milk composition (%)	Urban	Peri-urban	Rural
Fat	3.79 ± 0.09	3.79 ± 0.12	4.49 ± 0.43
Protein	3.05 ± 0.063	3.2 ± 0.034	3.5 ± 0.41
SNF	8.2 ± 0.15	8.2 ± 0.33	8.56 ± 1.56
Density	29.9 ± 0.29	30.5 ± 0.29	31.15 ± 1.65
Lactose	4.36 ± 0.9	4.66 ± 0.1	4.34 ± 1.43

1 $$\mathrm{SNF\%}= \left(\frac{\mathrm{milk\, density }(\mathrm{Kg}/\mathrm{L})}{4}\right)+\left(0.22*\mathrm{BF}\left(\mathrm{\%}\right)\right)+0.72$$

The milk energy content (ECM) was calculated using the following equation [41]:

2 $$\begin{aligned}\mathrm{ECM}\left(\mathrm{MJ}/\mathrm{kg}\right)&= 0.0386\mathrm{BF}\left(\mathrm{g}/\mathrm{kg \,milk }\right)\\ &\quad+0.0205\mathrm{ SNF }\left(\mathrm{g}/\mathrm{kg \,milk}\right)-0.236\end{aligned}$$

#### Feed characterization and seasonal diet composition

A recent carbon footprint study indicated that the IPCC Tier II approach is sensitive to changes in input parameters such as methane conversion factors (Ym) and diet digestibility (DMD) [5, 42]. The data on seasonal feed types and feed proportions were collected on the farm, and seasonal weighted DMD values were computed to reduce uncertainty in the assessment of Emission Factors (EF) and to account for seasonal feed baskets (Additional file 1: Table S1). We first identified the common feed resources used for dairy cattle and grouped them into six major types in the study area, namely: natural pasture (grazing), pasture hay, crop residue and pulse hulls, compound dairy ration (concentrate), bran, middling, and other cereal grain mill by-products, oil seed cakes and meals, agro-industrial by-products, local brewery residues (Table 4). The detailed diet components are presented in Additional file 1: Table S1. The digestibility of these feedstuffs was derived from previously published work and extensive characterization of livestock feed resources in the study area (Feyissa et al. [38]) (Table 2). The seasonal DMD content and the proportion of feed given to the different categories of animals in the farming systems were considered in our analysis. The average weighted seasonal value of digestible energy was estimated for the wet season (9 months) and dry season (3 months) [43]. Accordingly, the DMD of natural pasture, forage crops, and crop residues during the dry and wet seasons were estimated, and the weighted average was used to calculate EFs.

**Table 2:** Dry matter digestibility (DMD %) of the major feed available in the study area

Feed type	Season		References
	Wet	Dry	
Natural pasture (Native)	58.49	49.50	[54–57]
Grass hay	59.3	51.91	[55, 58, 59]
Commercial concentrate	72.28	72.28	[59]
Wheat bran	71.15	71.15	[59]
Wheat middling	79.03	79.03	[59]
Oats grain (*Avena sa*t*iva*)	76.03	76.03	[59]
Noug cake	70.99	70.99	[59]
Line sead cake	72.19	72.19	[59]
Cotton seed cake	84.67	84.67	[59]
Desho grass (*Pennisetum glaucifolium*)	52.68	49.36	[60, 61]
Napier grass (*Pennisetum purpureum*)	50.5	47.87	[62]
Vetch (*Vicia species*)	66.47	66.47	[63]
Alfalfa	76.63	76.63	[64]
Oats straw	46.89	46.19	[62]
Wheat straw	41	39.12	[56, 59]
Barley straw	41.32	41.32	[58, 65]
Teff straw	46.01	44.36	[56, 66]
Grass pea hull	61.42	61.42	[59]
Chickpea straw	50.16	50.16	[56, 59]
Faba bean hull	61.42	61.42	[65, 67]
Brewery residues	69.46	69.46	[59]
Atela (a local brewery)	73.66	73.66	[59]

In rural farms, cattle herds spend most of their time grazing on stubble after harvest and during the dry season, when natural pasture is depleted. The proportion of crop residues in the total diet increases in the dry season compared to the wet season (Table). To simulate the seasonal diet composition and the average apparent DMD values, the percentage of each feedstuff and their nutritional values were used. Finally, the calculated weighted value of DMD was used to estimate EF for each category and farming system. Feed digestibility was calculated for each feed, season, and farming system using an equation developed by Oddy et al. [44].

3 $$\begin{aligned}\mathrm{DMD }\left(\frac{\mathrm{g}}{100}\mathrm{g DM}\right) & = 83.58- 0.824 *\mathrm{ ADF }\left(\frac{\mathrm{g}}{100}\mathrm{g DM}\right)\\ &\quad+ \left(2.626 *\mathrm{ n }\left(\frac{\mathrm{g}}{100}\mathrm{g DM}\right)\right)\end{aligned}$$

#### Activity data

The IPCC emphasized the importance of animal feeding conditions in estimating the net energy dissipated by animals for grazing [16]. However, default values for the coefficient of activity (Ca) corresponding to the animal's feeding situation were provided in the IPCC guidelines without a detailed description of the feeding situation. Hence, Ca was estimated using the equation given in NRC [45] and the East African dairy methodology [32]. Due to relatively high population density and the expansion of crop production, grazing cattle are mostly kept in small private pastures and on the roadside, and expend very little energy to obtain feed. The average grazing distance (2–3 km) was estimated using survey data and triangulated using secondary data from East Africa [46, 47].

The National Research Council (NRC) classifies the net energy required for activity into two components: energy requirements for walking and energy requirements for grazing or eating activity [45]. According to NRC [45], the energy associated with eating is 0.0012 MJ/kg body weight; walking in a flat area is 0.00045 MJ/kg per km; and cows grazing on hilly terrain is 0.006 MJ/kg per km.

Where the net energy requirement for maintenance ${(NER}_{m})$ was calculated using IPCC [14] Eq. 10.3, megacalories (Mcal) were converted to MJ by multiplying by 4.20.

Based on the above premise, Ca was evaluated for different animal categories.

4 $$\begin{aligned} & \mathrm{Annual\, average\, km\, walked\, per \,day }\\ &\quad= \left(km \,in \,wet \,season*(months \,of \,wet \,season/12\right)\\ &\quad\quad+ \left(km\, in \,dry \,season*(months\, of \,dry\, season/12\right)\end{aligned}$$

If the proportion of feed obtained from grazing per day > 0, then C_a_ is calculated as follows

5 $${C}_{a}=\frac{\left(0.00045*LW*annual\, average\, km\, per \,day\right)+\left(0.0012*LW\right)*4.1868}{{ NE}_{m}}$$

The energy cost for cows grazing hilly topography is higher than that for cows grazing relatively flat pastures, so the energy requirements for maintenance increased by 0.006 Mcal of NERm/kg body weight. To convert ME to ${NER}_{m}$, an efficiency of 0.7 was used [48].

6 $$\begin{aligned} C_{a} \,& = \,{{\left( {\left( {0.00045\, * \,annual\,average\,km\,per\,day} \right)\, }\right.}} \\ & \quad {{\left.{ + \,\left( {0.0012\, * \,LW} \right)\, }\right.}} \\ & \quad {{\left.{+ \,\left( {0.006\, * \,BW} \right)\, * \,4.1868} \right)} \mathord{\left/ {\vphantom {{\left( {\left( {0.00045\, * \,annual\,average\,km\,per\,day} \right)\, + \,\left( {0.0012\, * \,LW} \right)\, + \,\left( {0.006\, * \,BW} \right)\, * \,4.1868} \right)} {NER_{m} }}} \right. \kern-0pt} {NER_{m} }} \end{aligned}$$

In Ethiopia, energy expenditure for traction or plowing is not well documented. As a result, an estimate of work hours was collected during the survey for the current study. Accordingly, in rural smallholder farming systems, oxen are assumed to plow for 3.5 months of the year (not including Sundays), 7.5 h per day, and thresh for 1 month (not including Sundays and other holidays). Values for energy expenditure from traction or plowing were calculated using Lawrence and Stibbards [49], who suggest an energy expenditure for walking of 2.1 MJ/kg LW and work efficiency for plowing of 0.3 for Brahman cattle. Cattle maintain traction efforts equivalent to 12% of their LW at a speed of 0.6–1.0 m/s, which indicates an additional energy expenditure of 0.4j/m/kg LW [50]. Hence, according to Marquardt, plowing requires (at 0.8 m/s velocity) 0.002 MJ/h/kg LW [51]. Thus, energy expenditure from plowing was calculated as follows:

$$\begin{aligned} MER_{P} \,\left( {{\text{MJ}}} \right)\, & = \,{\text{Work}}\,{\text{hours}}\,\left( {{{\text{h}} \mathord{\left/ {\vphantom {{\text{h}} {\text{d}}}} \right. \kern-0pt} {\text{d}}}} \right)\, \\ &\quad* \,Days_{work} \, * \,{\text{MLW}}\,\left( {{\text{kg}}} \right)\, \\ &\quad* \,0.002\,\left( {{\text{MJ}}} \right) \end{aligned}$$

### Estimation of total energy expenditure of animal

The total energy expenditure for each animal (category) was calculated by adding the metabolizable energy requirements (MER) for maintenance ${(MER}_{M})$, growth ${(MER}_{G})$, lactation ${(MER}_{L})$ for lactating animals, waking ${(MER}_{W})$, and plowing/traction ${(MER}_{P})$, if applicable. Energy expenditure was calculated by using equations derived from CSIRO [48].

Estimation of energy requirements for maintenance ${(MER}_{M})$

7 $$\begin{aligned} & MER_{M} \,\left( {{{MJ} \mathord{\left/ {\vphantom {{MJ} {day}}} \right. \kern-0pt} {day}}} \right)\, \\ &\quad= \,K * \,S\, * \,M\,\\ & \qquad\left( {0.26\, * \,LW^{0.75} \, * \,\frac{{\exp \,\left( { - 0.03A} \right)}}{{\left( {0.02\, * {M \mathord{\left/ {\vphantom {M D}} \right. \kern-0pt} D}} \right)\, + \,0.5}}} \right) \end{aligned}$$

where: K = 1.3 (the intermediate value for *Bos Taurus*/*Bos indicus*).

S = 1 for female.

M = 1.15 for male.

LW = Mean live weight for each season. However, live weight lost during the dry season is expected to be compensated in the wet season, with no weight loss or gain for adult animals [43].

A = age in years.

M/D = metabolizable energy content (ME MJ/DM KG) where;

8 $$\frac{{\text{M}}}{{\text{D}}}\, = \,0.172\,{\text{DMD}}\, - \,1.707$$

Estimation of energy requirements for Growth ${(MER}_{G})$

9 $${MER}_{G} (\mathrm{MJ}/\mathrm{d}) =(\frac{{LW}_{chage}*0.092*EC}{0.043*M/D})$$

where: EC (MJ/kg) = energy content of the tissue (18mj/kg) [38]

Estimation of energy requirements for lactation (MER_L_)

10 $${\mathrm{MER}}_{L}\left(\frac{MJ}{day}\right)= \left[\left(MY\left(L\right)\right)*ECM(MJ))/((0.02*M/D)+)+0.4\right]$$

$${MER}_{L}= \frac{(DMY*ECM)}{(\left(0.02*\frac{M}{E}\right)+0.04)}$$

where:

11 $${\text{MY~}}\left( {\frac{{\text{l}}}{{\text{d}}}} \right) = {\text{~}}\frac{{Total~milk~recorded~per~season~\left( L \right)}}{{Number~of~days~~in~season~\left( L \right)}}\, + \,DCMC~\left( L \right)$$

ECM (MJ/kg) = Energy content of milk MJ/kg.

Milk consumed by pre-ruminant calves (DCM_L_) was estimated following Radostits and Bell [48]. The growth rate of (0.340 and 0.362 kg/d) for high grade and the multipurpose breed was taken and calculated DCM as follows:

12 $$\mathrm{DCM}(\mathrm{L})= {(LW}_{Calves}\left(kg\right)*0.107L/kg)+(154L/0.1kgLWG)$$

Estimation of energy requirements for walking/grazing ${(MER}_{W})$

13 $${MER}_{T}(MJ/day)=Dist(km)* LW(KG)* 0.0026$$

where: DIST = average distance covered.

LW = live weight.

0.0026 = the energy expended (MJ/LW kg).

Estimation of energy requirements for plowing ${(MER}_{P})$

14 $$\begin{aligned} \left( {MER_{P} } \right)\,\left( {{\text{MJ}}} \right)\, & = \,{\text{Work}}\,{\text{hours}}\,\left( {{{\text{h}} \mathord{\left/ {\vphantom {{\text{h}} {\text{d}}}} \right. \kern-0pt} {\text{d}}}} \right)\, \\ &\quad* \,{\text{Daywork}}\, * \,{\text{MLW}}\,\left( {{\text{kg}}} \right)\, \\ &\quad* \,0.002\,\left( {{\text{MJ}}} \right) \end{aligned}$$

The daily total energy expenditure (MER_Total_) for each animal category in each production system/season was then calculated as:

15 $$\begin{aligned} & MER_{Total} \,\left( {{{{\text{MJ}}} \mathord{\left/ {\vphantom {{{\text{MJ}}} {{\text{day}}}}} \right. \kern-0pt} {{\text{day}}}}} \right)\, \\&\quad= \,MER_{M} \, + \,MER_{G} \, + \,MER_{L} \,\\ &\quad\quad + \,MER_{A} \, + \,MER_{P} \end{aligned}$$

#### Computation of daily methane production (DMP) and emission factor (EF)

The daily methane production (DMP) was estimated as a factor of dry matter intake DMI [52].

16 $${\text{DMI}}\,\left( {{\text{kg}}/{\text{d}}} \right)\, = \,\frac{{{\raise0.7ex\hbox{${MER_{Tootal} \,\left( {MJ/day} \right)}$} \!\mathord{\left/ {\vphantom {{MER_{Tootal} \,\left( {MJ/day} \right)} {\left( {GE \left( {MJ/kg} \right)*\left( {DMD/100} \right)} \right)}}}\right.\kern-0pt} \!\lower0.7ex\hbox{${\left( {GE \left( {MJ/kg} \right)*\left( {DMD/100} \right)} \right)}$}}}}{0.81}$$

where: GE = gross energy of the diet assumed to be 18.1 MJ/kg DM.

0.81 = the factor to convert metabolizable energy to digestible energy.

Then the estimated DMI was used to calculate DMP by using an equation developed by Charmley et al. [48]

17 $$DMP \left(g\right)=20.7*DMI (kg/day)$$

The mean DMP for each class of animal was calculated. This was then used to calculate annual enteric methane EF (CH_4_ kg/head/year):

18 $$EF= \frac{\left({DMP}_{Wet season}+{DMP}_{Dry season}\right)*365}{2*1000}$$

19 $$\mathrm{EI}=\frac{\mathrm{DMP}}{FPCM}$$

where EI = Emission Intensity.

DMP = Daily methane production, g CH_4_/day.

FPCM = Milk yield in fat and protein corrected milk (kgFPCM = MY*1.031) [53]

EI_CO2-eq_ = (DMP/FPCM*1000)*34 (to convert to kg and carbon equivalenct).

Where: EI_CO2-eq_ = emission intensity, kg CO_2_-eq kg^−1^ FPCM.

Here, emission intensity expressed in kg CO_2-_eq per kg FPCM.

### Data analysis

Descriptive statistics and one-way ANOVA were used to analyze quantitative data. The one-way ANOVA was used to examine the variation in EF, emission intensity (EI), %DMD, and DMP among the three farming systems, while the post-hoc test (Tukey tset) was used to compare means. Throughout the entire document, we have used the value for dult female (dairy cattle) for consistency and to compare our findings with the body of current knowledge. The Statistical Package for Social Studies (SPSS) (2003) version 26 and the Microsoft Excel computer programme were used to conduct the analyses. A Monte Carlo simulation (MCS) was used to estimate the uncertainties in the enteric methane emissions factor across the three farming systems using the IPCC methodology. The margin of error (MOE) with a 95% confidence interval was used to estimate uncertainty. The margins of error were calculated with a z-score of 1.96 and a value of 0.05. The contribution of each variable to total uncertainty was calculated using Spearman's ranked correlation coefficients (Additional file 1: Table S2).

## Results

### Farming system characteristics

Three types of smallholder dairy farming systems were identified and characterized (Table 3). Cattle were categorized into five classes based on age and sex. Herd composition varied across farming systems, and the largest female cattle were reported in the urban farming system. All cows in urban and peri-urban farming systems were either pure exoticbreeds or cross breeds with medium to high exotic blood levels (the cross of Holstein and *Bos-indicus*). Dual-purpose indigenous cattle were dominantly kept in rural farming systems. A significant (P < 0.05) variation was reported in the average daily milk yield across different farming systems (Table 3) [38]. The average daily milk yield per cow was significantly higher in urban than peri-urban and rural farming systems. Similarly, all cattle categories in rural farming systems had lower LW than in peri-urban and urban farming systems.

**Table 3:** Herd structure, breed/genotype composition, mean live weight, (kg ± SD), and animal performances for the three farming systems in Selale milkshed

Farming system	Urban		Peri-urban		Rural	
Breed & genotype	Pure/high-grade		High-grade		Local zebu	
	Mean ± SD	Observation	Mean ± SD	Observation	Mean ± SD	Observation
Adult dairy cows	429.2 ± 43.7^a^	508	423.3 ± 42.0^a^	545	294.2 ± 34.5^b^	159
Adult male	435.3 ± 47.3	10	425.3 ± 57.2	32	351.1 ± 28.3	84
Growing female	271.4 ± 44.0	270	265.7 ± 39.9	352	198.3 ± 42.9	56
Growing male	288.1 ± 55.9	25	280.1 ± 45.9	33	234.6 ± 17.5	34
Calf (≤ 12 m) male and female	141.2 ± 25.0	144	134.4 ± 25.9	144	113.4 ± 14.4	76
Calf (≤ 6 m) male and female	75.1 ± 11.2	241	72.6 ± 12.0	220	57.5 ± 7.3	130
Breeding bull	NA		NA		346.3 ± 38.5	54
Fattening	NA		NA		375.7 ± 35.5	54
Herd performance						
MY L/day	10.4 ± 0.9^a^		9.0 ± 1.1^b^		1.8 ± 0.2^c^	
ECM (MJ/KG)	3.0 ± 0.6^a^		3.0 ± 0.6^a^		3.7 ± 0.5^b^	
Working hrs/day			7.80 ± 1.0		7.99 ± 0.8	

Adult cows (≥ 3 years); Adult male (≥ 3); growing female (1–3 years); growing male (1–3 years); Calve (≤ 1); calve (≤ 6 m). Means with different superscript letters in the same row indicate significant differences at p < 0.05, if not it indicate non-significant

*ECM* energy content of milk, *ADWG* average daily weight gain, *MY* milk yield per day, *NA* not applicable, *Working hrs* Working hours per day for draught oxen, *SD* Standerd deviation

### Diet composition

Table 4 shows feed proportion and estimated values for dry matter digestibility (% DMD). Natural pasture hay, crop residues, and pasture grazing constitute the dominant sources of livestock feed in the study area. Wheat bran, wheat middling, and noug (*Guizotia abyssinica*) seed cake were the most commonly used agro-industrial by-products, with oat grain/hull and grass pea hull serving as local supplements. The share of these feed ingredients in animal diets varied across farming systems and seasons of the year. On the urban farm, all animals were stall-fed, whereas in the per-urban farming system, stall-feeding with limited grazing on restricted areas and private pasture was the common feeding practise. On the other hand, grazing is the main feeding system throughout the year for rural farming systems, with a minimum supplementation for adult females. Our estimated value of the weighted mean DMD of the feed basket varied among farms and seasons of the year. The DMD% of the total diet was higher (P < 0.05) in the urban smallholder dairy farm, followed by the peri-urban dairy farm, and the least in the rural smallholder dairy farms.

**Table 4:** Estimated major feed resources and its proportion and weighted digestible values (DMD%) based on the three farming systems in the Selale milkshed

Feed proportion (%)						
Feed type	Urban		Peri-urban		Rural	
Season	Wet season	Dry season	Wet season	Dry season	Wet season	Dry season
Pasture grazing	NF	NF	10.0	3.00	52.0	40.0
Grass hay	37.0	33.0	23.0	24.0	4.00	8.00
Crop residues and hulls	19.1	16.42	24.02	28.5	24.5	31.50
Cultivated forage	1.42	1.00	4.92	3.50	6.00	2.50
Compound dairy ration- concentrate	4.00	4.50	3.00	3.50	NF	NF
Bran, middling & other cereal grain mill by-products	19.5	20.0	16.0	18.0	7.00	8.50
Oil seed cake and middling	13.0	16.0	11.0	11.0	NF	2.00
Agro-industrial by-product	6.00	8.10	7.00	7.00	3.50	4.50
Local beverage residue (Atela)	NF	1.00	1.00	1.50	3.00	3.50
Diet DM digestibility %	62.58 ± 3.44^a^	61.89 ± 3.93^a^	60.69 ± 3.25^b^	59.82 ± 2.57^b^	57.46 ± 3.83^c^	54.08 ± 3.82^d^
Average DMD % (Mean ± SD)	62.3 ± 3.44^a^		60.3 ± 3.25^b^			55.8 ± 3.8^c^

Means with different superscript letters in the same row indicate significant differences between seasons of the year at (P < 0.05)

*DMD* dry matter digestibility, *SD* standard deviations, *NF* not fed

### Daily methane production, emission factor, and emission intensity

Metabolizable energy requirements (MER_T_) for different categories of animals across farming systems are presented in Table 5. Metabolizable energy requirement for lactation **(**MER_L_) was the largest component of MER for adult females in urban and peri-urban farming systems, whereas metabolizable energy requirement for maintenance (MER_M_) was the major component of MER for all classes of cattle in the rural farming systems.

**Table 5:** The weighted mean annual metabolizable-energy requirements (MER, MJ/day) in Selale milkshed

Farming systems	Urban				Peri-urban						Rural					
Animal categories	MER_M_	MER_G_	MER_L_	MER_T_	MER_M_	MER_G_	MER_L_	MER_W_	MER_P_	MER_T_	MER_M_	MER_G_	MER_L_	MER_W_	MER_P_	MER_T_
Adult female	37.29	NA	51.33	88.62	37.24	NA	44.61	2.76	NA	84.62	27.81	NA	11.56	1.60	0.0	40.97
Adult male	36.75	NA	NA	36.75	36.83	NA	NA	2.80	13.12	52.74	36.35	NA	NA	1.93	5.54	43.82
Growing female	27.79	13.90	NA	41.69	27.33	14.36	NA	1.74	NA	43.43	22.39	13.32	NA	1.10	NA	36.81
Growing male	29.07	15.18	NA	44.25	28.44	15.16	NA	1.83	NA	45.43	25.38	13.55	NA	1.3	NA	40.24
Calf (≤ 12 m)	17.58	16.98	NA	34.58	17.15	16.84	NA	0.88	NA	34.87	15.59	15.42	NA	0.64	NA	31.65
Calf (≤ 6 m)	11.13	19.54	NA	30.67	10.96	17.26	NA	NA	NA	28.23	7.95	16.95	NA	NA	NA	24.88
Fattening male	NA	NA	NA	NA	NA	NA	NA	NA	NA	NA	36.03	NA	NA	2.29	6.55	44.87

There were either no breeding bull and fattening male animals or few in urban and peri-urban farming systems

*NA* not applicable, *MER*_*P*_ is not applicable in urban faming systems

Daily methane production (DMP) for different categories of cattle is presented in Table 6. DMP varies considerably across smallholder farming systems. DMP and EFs showed significant differences among the different production systems, with the highest (P < 0.05) DMP and EF observed in urban farming systems and the lowest (P < 0.05) in rural farming systems, with an intermediate value in the peri-urban dairy production systems (Table 8).

**Table 6:** Daily methane production (DMP) (kg methane (CH_4_)/head/day) for different categories of animal across farming systems

Farming system	Adult female	Adult male	Growing female	Growing male	Calf ≤ 1r	Fattening
Urban	199.21	82.60	93.72	99.47	77.70	NA
Peri-urban	190.21	118.56	97.62	102.12	78.39	NA
Rural	92.09	98.51	82.76	90.45	71.15	100.86

*NA* Not applicable

The estimated EFs were higher (72.71 and 69.43 kg per head per year) for lactating cows, followed by growing males in urban and peri-urban farming systems, respectively (Table 7). In contrast, the largest EF was reported for fattening males, followed by adult females in rural farming systems. This is most likely due to the large body weight of fattening animals and the metabolic energy requirements for plowing.

**Table 7:** Emission factors (mean ± sd., kg CH_4_ /head/year) for different categories of cattle

Farming systems	Emission factors (kg CH_4_ /head/year)					
	Adult female	Adult male	Growing female	Growing male	Calf (≤ 1)	Fattening male
Urban	72. 71 ± 6.24^a^	30.15 ± 2.43^a^	34.21 ± 2.71^a^	36.31 ± 2.78^a^	26.67 ± 2.3^a^	NA
Peri-urban	69.43 ± 5.59^b^	43.28 ± 3.29^b^	35.63 ± 3.14^b^	37.27 ± 3.12^a^	25.89 ± 2.25^a^	NA
Rural	33.68 ± 2.86^c^	35.92 ± 2.84^a^	30.2 ± 2.61^c^	33.01 ± 2.46^b^	23.2 ± 2.1^b^	36.82 ± 3.23
Mean	64.01 ± 5.23	37.84 ± 2.81	36.39 ± 3.18	38 .01 ± 3.16	26.5 ± 2.2	

Different superscript letters in the same column indicate significant differences between farming systems (P < 0.05)

The EI (CO_2-_eq kg^−1^ MY) was highest (P < 0.05) in rural and lowest (P < 0.05) in urban farming systems, with an intermediate value in the peri-urban dairy farming systems (Table 8). Emission intensity (CO_2-_eq kg^−1^ MY) was significantly higher in rural than in peri-urban and urban farming systems.

**Table 8:** Daily methane production (g/day), emission factors (mean ± sd., kg CH_4_ kg/head/year), and emission intensity (CO2-eq kg^−1^ MY) for adult female

Farming system	Urban	Peri-urban	Rural
MY(kg FPCM)	10.40 ± 0.84^a^	8.95 ± 1.11^b^	1.77 ± 0.18^c^
DMP (g/day)	199.2 ± 26.2^a^	190.21 ± 25.99^b^	92.9 ± 12.03^c^
EF (CH_4_ kg/head/year)	72. 71 ± 8.31^a^	69.43 ± 7.51^b^	33.61 ± 3.17^c^
EI (CO2-eq kg^−1^MY)	0.6432 ± 0.10^a^	0.71 ± 0.10^b^	1.78 ± 0.23^c^

Different superscript letters in the same row indicate significant differences between farming systems (P < 0.05)

*FPCM* Fat and protein corrected milk

The uncertainty of CH_4_ emission factors for adult dairy cows was ± 21%, 19%, and ± 16.0% in urban, peri-urban, and rural dairy farming systems, respectively (Table 9).

**Table 9:** Uncertainties in enteric methane emission factors

Farming system	Cattle categories					
	Adult female (%)	Adult male (%)	Growing female (%)	Growing male (%)	Calf (≤ 1) (%)	Fattening male (%)
Urban	20.1	17.1	31.7	22.6	34.5	
Peri-urban	19.4	19.4	29.4	21.7	37.5	
Rural	16.7	15.5	31.3	14.6	27.9	24.1
Mean	19.6	16.9	30.95	16.3	31.9	24.1

## Discussions

### Seasonal feed digestibility

The variation in seasonal feed baskets and DMD of the feed among rural smallholder farmers could be due to a year-round reliance on natural pasture and crop residues. This might be attributed to the proportion of various feed types in animal diets across farming systems and seasons. In rural farming systems, for instance, crop residue and green grass from natural pasture constituted 90% of the rural SHF's total feed basket throughout the year. Feyissa et al. [54] reported that the mean crude protein (CP) contents of natural pasture were reduced by 30.2%, while both IVOMD and ME contents were reduced by 17.8% each with the delay in harvesting time from mid-October (full heading stage of the pasture) to late-November (one and a half months past the full heading stage). Similar trends in digestible energy per season have been reported in Kenya by Goopy et al. [68] and in Senegal by Ndao et al. [47]. Modest seasonal DMD variation was reported in the urban and peri-urban farming systems, owing to the high digestibility of concentrate-based diet supplementation in the daily dry matter intake. The weighted mean DMD of the feed basket estimated in the present study is similar to the studies of Wassie et al. [5] and Goopy et al. [68] in Ethiopia and Kenya, respectively, but is greater than the IPCC default estimate for Africa [69].

### Emission factors and uncertainties

Metabolisable energy requirements for lactation (MER_L_) were the most important constituents of the metabolizable energy requirement, followed by MER_M_ in intensive (urban and peri-urban) smallholder farming systems, whereas MER_M_ was the largest constituent in extensive (rural) farming systems. The considerable variation in MER_L_ across the farming systems is attributed to significant differences in milk yield and feed intake between the farming systems. This might be because production influences the energy demand of adult female cows and their high feed intake, thereby increasing enteric CH_4_ production. Since cattle productivity in rural farming systems is low, a large proportion of gross energy intake is used for maintenance and is affected by live metabolic weight [70]. Previous studies in Sub-Saharan Africa have found similar results [46, 47, 68]. The significantly lower (P < 0.05) estimated DMP and EF for adult females in rural smallholder dairy farming systems than in urban and peri-urban farming systems could be due to the variation in production performances (milk yield, live weight, weight gain) and thus differences in energy requirements for maintenance and production across farming systems (Table 8). For example, mature females in urban and peri-urban farming systems were heavier and had more milk yield, which led to higher EF than in rural farming systems. Similarly, the observed variation could also be attributed to low feed intake due to body weight differences, feed characteristics, and breed differences. Consistent with the current observations, Jo et al. [28] and Shibata and Terada [27] indicated that variation in livestock production systems (feed quantity and quality, body weight, feeding level, and livestock activity and health) has a wide range of impacts on enteric CH_4_ emissions. Parta [71] has also established a significant link between intake and methane production.

The average uncertainty of emission factors for adult dairy cows found in this study is comparable with the default uncertainty range for Tier II emission factors [14] and the report of Wassie et al. [5] in Ethiopia but less than Wilkes et al. [72] in Kenya. Feed digestibility, live weight, and work hours had considerable influences on enteric CH_4_ emission factors for adult female and adult male cattle, respectively (Table 9). Due to a lack of precision in seasonal body weight estimation using a heart girth meter, seasonal weight gain and loss for adult cattle were not considered. Hence, we assumed live weight lost during the dry season is expected to be compensated for in the wet season with no weight loss or gain for adult animals [43], which may contribute to the uncertainty of the EF estimates used. The digestibility of the main diet composition used in this study comes from secondary sources, which adds uncertainty to the estimates of the emission factor.

### Emission intensity

The significantly higher enteric methane EI per kg of FPCM and for adult females, in rural than in urban and peri-urban farming systems could be attributed to the lower average feed digestibility and crude protein content reported in rural than in peri-urban and urban farming systems. As previously stated, seasonal feed baskets have shown significant variation in quality and quantity among rural smallholder farming systems, which may be due to a reliance on natural pasture and crop residue throughout the year (Table 4). In rural farming systems, DMD and crude protein content were below the 12–18% crude protein requirements of productive dairy cows [73]. Low digestible feed is linked to high enteric CH_4_ emissions and, as a result, high EI per kg of milk yield [74, 75]. According to Garg et al. [9], feeding balanced rations increased the milk yield of dairy cows, which then helped reduce the EI of milk. According to Gerber et al. [75], Zehetmeier et al. [76], and Garg et al. [9], increasing milk yield from 1300 to 5000 kg of FPCM/cow/year results in a large reduction in EI. Zehetmeier et al. [76] and Garg et al. [9] stated that emissions per kilogram of milk yield decreased with increasing production intensity, with the highest values for low-input systems and the lowest values for intensive farming systems.

The variation in enteric methane EI across production systems in our findings indicates that smallholder dairy production systems, where dairy animal productivity is low to medium, offer a good opportunity to reduce EI while improving productivity by feeding a nutritionally balanced ration. Consistent with this, Garg et al. [9] and Rotz et al. [77] also indicated that dietary manipulation has a considerable potential for improving milk production and lowering GHG emissions from dairy animals. Because diet composition directly affects CH_4_ emissions, diet manipulation is the most direct, and arguably the most effective, method of lowering the GHG intensity of milk for intensive dairy operations [78]. Hence, increased animal productivity through improved feed offers important mitigation potential for smallholder dairy farming systems across intensification gradients in the study area.

### Comparisons with previous estimates

The variation between our results and some of the previous work cited earlier may in part be due to the approach they used and the value of input parameters (e.g., DMD, milk yield, body weight) used in predicting emission values (Table 10). For instance, our current estimate of average weighted EF for adult females is broadly consistent with the value in Sub-Saharan Africa, which provides an estimate in the range of 30–80 kg CH_4_/year [46, 47, 68]. Though they used a similar approach to estimate EF, the milk yield and the feed digestible energy are considerably lower than the assumed values in this study. Compared with IPCC's [16] default value for high-producing dairy cows and low-productive multipurpose dairy cows in Africa, which are assumed to have lower body weight, milk yield, and feed digestibility than in the present study, our EF estimate for lactating dairy cows in urban, peri-urban, and rural farming systems was lower. The present estimate of EFs is also much lower than that reported by du Toit [79] for intensive farming systems in South Africa, which might be explained by the larger live weights of cattle in South Africa. Du Toit [79] also used a different approach to estimate CH_4_ emissions. Similarly, the average EF calculated for lactating cows (multipurpose/other cows) in rural smallholder farming systems (34 kg CH_4_ per head/year) is lower than the value estimated by IPCC [16] for the low productivity system of smallholder farming systems in Africa. Furthermore, our EF estimate is lower than the IPCC [16] value for low productivity systems in Sub-Saharan Africa and 38.5% lower compared with the 57.87 kg CH_4_/head suggested by Wassie et al. [5] for other cattle in Ethiopia. Similarly, our estimated value of EF is 35% lower compared with the 60 kg CH_4_/head suggested by Tadesse et al. [19] for crossbred dairy cattle and 39% higher than the value reported by Defar et al. [18] for intensive production systems and extensive mixed crop-livestock production systems in Ethiopia. This is mainly because either the approach they use or the input data they generate to represent the complexity of the system might be the source of the variation. Herrero et al. [80] suggest that lower estimates of CH_4_ emissions from enteric fermentation are likely due to the use of more aggregated methods to calculate CH_4_ emissions. In addition, our findings show that the EFs of replacement males and females in urban and rural farming systems were much lower than those reported by the IPCC for intensive and extensive farming systems in sub-Saharan Africa (Table 10). A similar result was reported for cattle in high-and low-productivity systems in the Latin American region [81].

**Table 10:** A Comparison of our findings to IPCC default values and Wassie et al. [5]

Cattle category	IPCC [16] Tier I			Wassie et al. [5]			Urban farming systems (Present Findings)		
	LW (kg)	MY (L)	EF	LW (kg)	MY (L)	EF	LW (kg)	MY (kg)	EF
Farming system									
Adult cows	400	5.9	104	429	6.4	78	429.19	10.4	72.71
Adult male	450	–	87	419		47	435.27		30.15
Growing female	212	–	86	261		39	271.37.51		34.21
Growing male	237	–	83	261		41	288.13		36.31
Rural farming systems									
Adult cows	247	2.4	88	286	1.25	58	294	1.77	33.61
Adult male	382		104	343		57	351		35.95
Growing female	135		76	181		41	198		30.21
Growing male	202		87	227		54	234		33.01
Fattened male	225		64	281		52	375		36.82

Adult cows ≥ 3 years; Adult male ≥ 3 years; Growing female = 1–3 years; Growing male = 1–3 years; Calve ≤ 1

## Conclusion

In the present study, we use a more detailed analytical approach of the IPCC Tier II by using information such as estimation of energy intake, diet quality, milk yield, and body weight, avoiding relying on the assumption of ad libitum intake to estimate daily methane production, thereby EF and EI across the three smallholder farming systems. Based on this approach, which could better represent the complexity of the smallholder dairy farming system, we estimated EFs of 73 and 34 kg CH_4_ for intensive and extensive farming systems, respectively. Our estimates are up to 22.1% and 59.6 lower than the IPCC [16] Tier I estimates for dairy cows and other cows, respectively. This suggests that IPCC Tier I and other studies partly rely on Tier I default values and coefficients, which tend to overestimate emissions from smallholder farming systems. Methane emission intensity has shown significant variation across the intensification gradients. Rural dairy farming systems showed significantly higher emission intensity per kg of milk yield than urban and peri-urban dairy farming systems. This study suggests that increased animal productivity through improved feed offers important mitigation potential for smallholder dairy farming systems across intensification gradients in the study area. Using country-specific activity data to accurately characterize emissions will aid in explaining the spatial variation in emissions across countries and regions. The level of uncertainty can be reduced by improving data quality by measuring these key parameters more accurately as well as collecting data that represents different systems of interest.

### Supplementary Information

**Additional file 1: Table S1.** Estimated feed compositions and its proportion based on the three farming systems sourced from the study. **Table S2.** Margin of error and probability density function (PDF) used in uncertainty analysis. **Table S3.** Input parameters and coefficients used to estimate emission factors for enteric methane emissions.

## Data Availability

Data and models will be available upon request. The software used is available online to reviewers.
