# Supplementary material for: Enteric methane emission factors of smallholder dairy farming systems across intensification gradients in the central highlands of Ethiopia
Source: Carbon Balance Manag. 2023 Nov 29;18:23. doi: 10.1186/s13021-023-00242-0 (PMC10688001; doi:10.1186/s13021-023-00242-0)
Supplement: Supplementary file 1 — Additional file 1: Table S1. Estimated feed compositions and its proportion based on the three farming systems sourced from the study. Table S2. Margin of error and probability density function (PDF) used in uncertainty analysis. Table S3. Input parameters and coefficients used to estimate emission factors for enteric methane emissions. [file 13021_2023_242_MOESM1_ESM.docx]

**Additional table**

**Table S_1_**. Estimated feed compositions and its proportion based on the three farming systems sourced from the study.

| **Feed proportion (%)** | | | | | | |
| --- | --- | --- | --- | --- | --- | --- |
| **Feed type** | **Urban SHF** | | **Peri-urban SHF** | | **Rural SHF** | |
| **Season** | **Wet season** | **Dry season** | **Wet season** | **Dry season** | **Wet season** | **Dry season** |
| Pasture grazing | NF | NF | 10 | 3 | 52 | 40 |
| Grass hay | 37 | 33 | 23 | 24 | 4 | 8 |
| Compound dairy ration Concentrate | 4 | 4.5 | 3 | 3.5 | NF | NF |
| Wheat bran | 10.5 | 11 | 8 | 8 | 2 | 3 |
| Wheat middling | 7 | 7 | 6 | 7 | 2.5 | 2.5 |
| Oats grain | 2 | 2 | 2 | 3 | 2.5 | 3 |
| Noug cake | 8.5 | 9 | 6 | 5.5 | NF | 2 |
| Cotton seed cake | 1.5 | 2.5 | 2 | 1.5 | NF | NF |
| Linseed mill | 1 | 1.5 | 1 | 1.5 | NF | NF |
| Soybean | 2 | 3 | 2 | 2.5 | NF | NF |
| Desho grass | 1.42 | 1 | 2.42 | 2.5 | 2 | 1 |
| Napier grass | NF | NF | NF | NF | 1 | NF |
| Vetch (Vicia sativa) | NF | NF | 1.5 | 1 | 1.5 | 0.5 |
| Alfalfa | NF | NF | 1 | NF | 1.5 | 1 |
| Oats straw | 2 | NF | 2.52 | 2.5 | 1.5 | 3 |
| Wheat straw | 5.58 | 5 | 8 | 8.5 | 8 | 11 |
| Barley straw | 5 | 5.42 | 7.5 | 9 | 6 | 9 |
| Teff straw | 4 | 4.5 | 4 | 5 | 5 | 5 |
| Grass pea hull | 1 | NF | 1 | 2 | 2.5 | 1.5 |
| Faba bean hull | 1.5 | 1.5 | 1 | 1.5 | 1.5 | 2 |
| Brewery residues | 4.5 | 5.58 | 5 | 4.5 | 1.5 | 2 |
| Molasess | 1.5 | 2.5 | 2 | 2.5 | 2 | 2.5 |
| Local beverage residue (Atela) | NF | 1.00 | 1 | 1.5 | 3 | 3.5 |

DMD = dry matter digestibility; SD = standard deviations; NF: not fed

**Table S_2_.** Margin of error and probability density function (PDF) used in uncertainty analysis

| Parameter | Farming systems | Uncertainties | PDF, justification | References |
| --- | --- | --- | --- | --- |
| Live Weight | Urban | ±22.0% -24.3% | Norma, SE small compared with mean | Calculated from the study |
|  | Peri-urban | ±21.2% -29.0% | Norma, SE small compared with mean | Calculated from the study |
|  | Rural | ±19.61%-34.5 | Norma, SE small compared with mean | Calculated from the study |
| Milk yield | Urban | ±16.96% | Norma, SE small compared with mean | Calculated from the study |
|  | Peri-urban | ±23.96% | Norma, SE small compared with mean | Calculated from the study |
|  | Rural | ±21.78% | Norma, SE small compared with mean | Calculated from the study |
| ECM | Urban | ±7.56% | Norma, SE small compared with mean | Calculated from the study |
|  | Peri-urban | ±7.56% | Norma, SE small compared with mean | Calculated from the study |
|  | Rural | ±6.49% | Norma, SE small compared with mean | Calculated from the study |
| DMD | Urban | ±10.82% | Triangular distribution minimum | Calculated from the study |
|  | Peri-urban | ±10.56% | Triangular distribution minimum | Calculated from the study |
|  | Rural | ±13.45% | Triangular distribution minimum | Calculated from the study |
| Work hours | Urban | NA |  |  |
|  | Peri-urban | ±23.79% | Norma, SE small compared with mean | Calculated from the study |
|  | Rural | ±19.25 | Norma, SE small compared with mean | Calculated from the study |
| CH_4_ conversion factor | Urban | ±20% | Norma, SE small compared with mean | IPCC, 2019 |
|  | Peri-urban | ±.20% | Norma, SE small compared with mean | IPCC, 2019 |
|  | Rural | ±20% | Norma, SE small compared with mean | IPCC, 2019 |

**Table S_3_**. Input parameters and coefficients used to estimate emission factors for enteric methane emissions

| **Parameter** | **Symbol** | **Unit** | **Sources** |
| --- | --- | --- | --- |
| Herd structure |  | % | The present study |
| The coefficient for estimating net energy | Cfi | MJ day/kg | IPCC, 2019 |
| Coefficient for activity | Ca | MJ day/kg | The present study |
| Methane conversion rate | Ym |  | The present study |
| Dry Matter Digestibility | DMD | (g/100)g DM | Seconder source |
| Metabolizable energy | M/D | MJ/DMkg | Estimated |
| Average live body weight for all categories | LW | kg | The present study |
| Average daily weight gain | ADG | kg | (Haile et al., 2011; Dadi et al., 2021) |
| Average daily milk yield | MY | Kg/day | The present study |
| Energy Content of Milk | ECM | MJ/Kg | The present study |
| Number of hours of work | Hour | hrs | The present study |
